# Determination of the oral carcinoma and sarcoma in contrast enhanced CT images using deep convolutional neural networks

**DOI:** 10.1038/s41598-025-06318-w

**Published:** 2025-07-01

**Authors:** Kritsasith Warin, Wasit Limprasert, Teerawat Paipongna, Sitthi Chaowchuen, Sothana Vicharueang

**Affiliations:** 1 Faculty of Dentistry, Thammasat University, Khlong Luang, Pathum Thani Thailand; 2 College of Interdisciplinary Studies, Thammasat University, Khlong Luang, Pathum Thani Thailand; 3 Sakon Nakhon Hospital, Mueang Sakon Nakhon, Sakon Nakhon Thailand; 4 Udonthani Cancer Hospital, Muang Udonthani, Udonthani Thailand; 5 StoreMesh, Thailand Science Park, Khlong Luang, Pathum Thani Thailand

## Abstract

Oral cancer is a hazardous disease and a major cause of morbidity and mortality worldwide. The purpose of this study was to develop the deep convolutional neural networks (CNN)-based multiclass classification and object detection models for distinguishing and detection of oral carcinoma and sarcoma in contrast-enhanced CT images. This study included 3,259 slices of CT images of oral cancer cases from the cancer hospital and two regional hospitals from 2016 to 2020. Multiclass classification models were constructed using DenseNet-169, ResNet-50, EfficientNet-B0, ConvNeXt-Base, and ViT-Base-Patch16-224 to accurately differentiate between oral carcinoma and sarcoma. Additionally, multiclass object detection models, including Faster R-CNN, YOLOv8, and YOLOv11, were designed to autonomously identify and localize lesions by placing bounding boxes on CT images. Performance evaluation on a test dataset showed that the best classification model achieved an accuracy of 0.97, while the best detection models yielded a mean average precision (mAP) of 0.87. In conclusion, the CNN-based multiclass models have a great promise for accurately determining and distinguishing oral carcinoma and sarcoma in CT imaging, potentially enhancing early detection and informing treatment strategies.

## Introduction

Oral cancer is a malignant lesion in the oral cavity and a life-threatening disease which is the 16th most common cancer worldwide, with approximately 390,000 new cases and half of the number of these new cases resulted in death in 2022^1^. Oral cancer can originate from epithelial tissues (carcinoma) or mesenchymal tissues (sarcoma). Both oral carcinoma and sarcoma present substantial challenges in oncology, contributing significantly to morbidity and mortality worldwide. These malignancies, affecting the oral cavity and surrounding structures, pose a considerable threat to global health due to their high incidence rates and potential for metastasis^2–4^. The diagnosis of oral carcinoma and sarcoma typically requires a combination of clinical examination, imaging studies, and tissue biopsy for histopathological examination, which is considered the gold standard for diagnosis. Imaging studies, such as computed tomography (CT) and magnetic resonance imaging (MRI), are used to evaluate primary tumors. CT scans, especially contrast-enhanced CT, are the gold standard for identifying tumors in the head-and-neck region and evaluation of any metastasis to nearby structures or distant organs, because of their increasing accessibility with a specificity range of 82%–100% and sensitivity range of 41%–82%^5^.

Treatment modalities for oral carcinoma and sarcoma share many similarities, with surgical removal of the tumor as the primary treatment, but may also have some differences depending on the characteristics of the tumors and their behavior. Surgical treatment of oral sarcomas may require more extensive surgical resection because of their aggressive nature, which tends to infiltrate surrounding structures. The prognosis for oral carcinoma and sarcoma depends on the stage and type of tumors^6,7^. Therefore, early, accurate and reliable screening is critical to improve the survival rate and quality of life of oral cancer patients.

In recent years, the use of deep convolutional neural networks (CNNs), a type of deep learning, to automate cancer detection (identifying the presence of cancer) and diagnosis (characterizing the cancer) in medical imaging has become increasingly common^8,9^. CNNs are particularly effective at identifying patterns in images to recognize objects, classes, and categories. CNN models have revolutionized various aspects of medical imaging and diagnostics, enhancing the detection and classification of cancerous lesions, including those in the oral cavity^10,11^. Various studies have shown that CNNs have been successfully applied to classify and detect head and neck lesions in CT images, yielding promising results^12^. The interpretation of these cancerous lesions in CT images can be complex and time-consuming, often requiring the expertise of experienced radiologists or surgeons. Given CNNs’ ability to identify complex patterns in CT images, their application could potentially distinguish the features of oral carcinoma and sarcoma, and be able to identify subtle differences of malignancy that might be missed by a clinician’s eyes, enabling more accurate screening and reducing human error in the interpretation process.

The aim of this study was to evaluate the performance of CNN-based multiclass classification and object detection models for distinguishing and detection of oral carcinoma and sarcoma in contrast-enhanced CT images. These models could aid clinicians to provide reliable diagnostic support, enhance decision-making as supplementary information in surgical planning, and ultimately improve the prognosis of oral cancer patients. The organization of this study is as follows.

- **Section 2: Materials and Methods—**This section describes the dataset, preprocessing techniques, dataset splitting strategy, class imbalance handling, and the configurations of the multiclass classification and object detection models used in this study.
- **Section 3: Results—**This section presents the study’s results, including performance metrics such as precision, recall (sensitivity), F1-score, specificity, accuracy, average precision (AP), mean average precision (mAP), and the area under the curve (AUC) of receiver operating characteristic (ROC) and precision-recall curves. A comparative analysis with state-of-the-art methods is also provided.
- **Section 4: Discussion—**This section interprets the results, explores their clinical application, and discusses the strengths of the models. Additionally, it addresses the limitations and potential areas for future work.
- **Section 5: Conclusion—**Finally, this section summarizes the key findings of the study, their clinical applications, and the significance of these findings in advancing the field of medical imaging using CNN techniques.

The major contributions of this study are as follows:

- **Advanced model application**: This study adopted state-of-the-art CNNs for multiclass classification and object detection, effectively distinguishing and identifying oral carcinoma and sarcoma in contrast-enhanced CT images. This demonstrates the models’ adaptability and efficacy in advancing medical imaging techniques.
- **Innovative data handling**: The study implemented a robust dataset handling procedure, including data splitting, and addressed class imbalance through data augmentation and resampling technique, minimizing overfitting and enhancing model accuracy.
- **Clinical relevance**: The study’s findings indicate significant potential for clinical application. The increased accuracy and robustness in detecting oral malignancies could facilitate early diagnosis and inform treatment planning, ultimately supporting improved patient care.

## Materials and methods

### Data acquisition

This study was approved by the Human Research Ethics Committee of Thammasat University (COA 011/2565) and was performed in accordance with the tenets of the Declaration of Helsinki. Informed consent for the study was waived by the Human Research Ethics Committee of Thammasat University because of the retrospective nature of the fully anonymized images. This study was performed under the guidelines of the Checklist for Artificial Intelligence in Medical Imaging (CLAIM) protocol^13^.

This study retrospectively analyzed the contrast-enhanced CT images data from 107 cases with a presentation of oral cancer in the cancer hospital and two regional hospitals from 2016 to 2020. The study data included 55 cases with the diagnosis of oral squamous cell carcinoma, and 52 cases with the diagnosis of oral sarcoma, including Osteosarcoma, B-cell lymphoma, and Clear cell sarcoma. All oral squamous cell carcinoma and sarcoma cases had a pathologic confirmation as a gold standard for the diagnosis. The contrast-enhanced CT images were captured using equipment from various manufacturers following standard imaging protocols. Two-dimensional planar reconstructions were carried out in the frontal, sagittal, and oblique planes, aligned with the long axis of the orbits, hard palate, and mandible. A rapid injection of 100 ml of Iopromide (Ultravist®, Schering AG, Germany) was used as the contrast medium. The CT images included various axial, sagittal, and coronal views with slice thicknesses of 1–2 mm in increments, and a matrix size of 512 × 512 pixels, with all patient information removed. The CT images were standardized to a consistent Hounsfield Unit (HU) range to minimize variability in tissue density representation. For developing the CNN-based classification and object detection models for oral cancer, the CT images selected for this study were in a two-dimensional axial view of the contrast-enhanced soft tissue window.

The total number of CT images was 3,259, which were selected from slices of CT image data of oral cancer patients that contained cancerous lesions and slices of CT images without pathology. Of these, 2,259 CT images of oral cancerous lesions were distributed to two classes, including oral carcinoma of 1,289 images, and oral sarcoma of 970 images. Another 1,000 normal CT images without pathology were selected from slices of CT images without pathologic lesions.

All CT images were uploaded to the Label Studio (Digital Storemesh, Bangkok, Thailand) server. Label Studio is a private web application for image annotation. To develop the CNN-based classification and object detection models, rectangular bounding boxes were drawn around each tumor lesion in CT images and labeled as “carcinoma” and “sarcoma” class according to the location, extension, and pathological reports of tumors by consensus of three oral and maxillofacial surgeons and one otolaryngologist with more than 5 years of experience in oral cancer surgery. For CT images without tumor lesions, all images were classified as “normal” class.

### Experiments

All images were randomly divided, and assigned to the training, validation, and test datasets using a 70:10:20 split, with a randomization by distribution to ensure an equal distribution of classes. The validation dataset was used to evaluate the model performance during training, while the test dataset was used to evaluate the model performance after training.

To address class imbalance within the dataset, we employed an integrated approach that included both image augmentation and resampling techniques. Image augmentation was performed using the imgaug library, which applies a series of random transformations to artificially increase the diversity of the training data. The augmentation techniques implemented included horizontal flipping, brightness adjustment, and channel shuffling, with specific emphasis on employing iaa.Fliplr(0.5), which flips images horizontally with a 50% probability. This strategy not only increases the effective size of the training dataset but also helps in achieving anatomical symmetry, thus preserving the structural consistency of the images. In conjunction with augmentation, we applied resampling techniques, combining oversampling of the minority class with undersampling of the majority class. This balanced class distribution was critical in ensuring the model did not develop a bias towards the majority class, which can compromise the performance in detecting less prevalent classes. These preprocessing techniques were pivotal in reducing overfitting and improving generalization, particularly in scenarios where the number of samples in the minority classes was limited. By enhancing the data available for training, we aimed to bolster the model’s robustness and predictive accuracy across all classes.

**Fig. 1:**
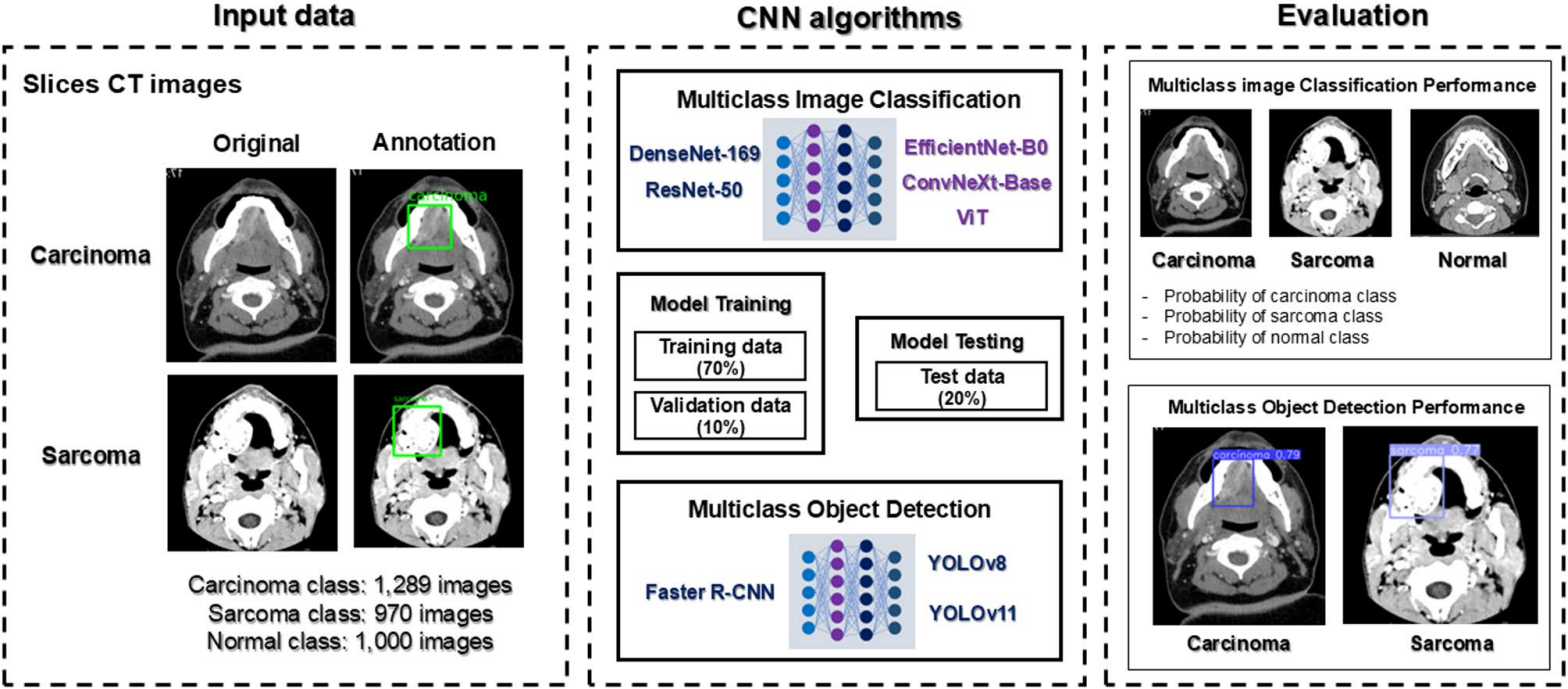
Workflow illustrating the dataset preparation, CNN algorithms, and evaluation process. *CNN* convolutional neural network, *CT* computed tomography.

This work was divided into two experiments by deploying well established CNN algorithms to create multiclass image classification and object detection models for the distinguish and detection of oral cancerous lesions in contrast-enhanced CT images. The workflow of these experiments is illustrated in Fig. 1.

### Multiclass image classification

This study incorporated various state-of-the-art multiclass classification CNN architectures to classify oral carcinoma and sarcoma in contrast-enhanced CT images. The architectures utilized include DenseNet-169, ResNet-50, EfficientNet-B0, ConvNeXt-Base, and ViT-Base-Patch16-224, each selected for their distinct capabilities and strengths to create CNN-based image classification models for multiclass classification (“carcinoma”, “sarcoma”, and “normal” classes).

Densely Connected Convolutional Networks (DenseNet) is a type of feed-forward CNN, which links each layer to every preceding layer. This architecture enhances learning efficiency by reusing features, thereby reducing the number of parameters and improving the progressive flow of information during training^14^.

Residual Network (ResNet) is a CNN architecture that incorporates residual units with identity mapping, enabling deep layers to learn directly from shallow layers and allowing the model to bypass one or more layers. This design aids in network convergence and allows ResNet to achieve higher accuracy in object classification by significantly increasing the network’s depth^15^.

EfficientNet-B0 employs a compound scaling method, harmoniously balancing network depth, width, and resolution, thereby providing efficient and powerful performance with fewer parameters. This makes it particularly suitable for efficiently handling the detailed patterns present in medical imaging^16^.

ConvNeXt-Base, inspired by the simplicity of Transformers and traditional convolutional networks, implements ConvNeXt blocks with streamlined design choices and fewer non-linearities, facilitating robust feature extraction with improved scalability and simplicity^17^.

The Vision Transformer (ViT-Base-Patch16-224) introduces a transformative approach by treating image analysis as a sequence modeling task, effectively capturing spatial relationships via patch embedding and transformer encoders. This model is especially adept at modeling complex data relationships, making it well-suited for handling the intricate features found in CT images^18^.

The images were preprocessed using a custom DataLoader implemented in PyTorch, an open-source deep learning framework. Each image was resized to 224 × 224 pixels, which is standard for compatibility with many deep learning architectures and balances making computational resources and maintaining image quality. We employed several state-of-the-art deep learning models, including DenseNet-169, ResNet-50, ConvNeXt Base, EfficientNet-B0, and ViT-Base-Patch16-224, all of which were pre-trained on the ImageNet dataset to leverage transfer learning. Optimization was conducted using the ADAM optimizer with learning rates adjusted to model complexity: 0.0002 for DenseNet-169 and ResNet-50 over 8456 iterations, and 0.000001 for ConvNeXt Base, EfficientNet-B0, and ViT-Base-Patch16-224 over 8600 iterations. A consistent batch size of 64 was used throughout. All coding was performed in Python with PyTorch, using a Jupyter Notebook on a Tesla V100 GPU, equipped with Nvidia driver 470.82 and CUDA version 11.4. This setup ensures robust computational support for the intensive training process.

### Multiclass object detection

The CNN-based object detection algorithms, Faster R-CNN, YOLOv8 and YOLOv11, were adopted to create the multiclass image object detection models for the detection of oral cancerous lesion in CT images as “carcinoma” and “sarcoma” class. Faster R-CNN is an object detection model that improves on Fast R-CNN by integrating a region proposal network (RPN) with the CNN. The RPN uses shared convolutional features from the full image with the detection network, making region proposals almost cost-free. This fully convolutional network simultaneously predicts object boundaries and objectness scores at every position^19^. You Only Look Once (YOLO) is a highly popular object detection architecture known for its exceptional accuracy and processing speed. It employs an advanced neural network architecture to predict bounding boxes and class probabilities simultaneously in an end-to-end manner^20^.

The images were preprocessed using custom loaders in PyTorch, supplemented by the Detectron2 framework, with input images resized to 256 × 256 pixels to suit the network configurations. Our models, including Faster R-CNN, YOLOv8, and YOLOv11, were pre-trained on ImageNet and COCO datasets, enhancing their capability to generalize on tumor detection in CT images. Training was conducted on an on-premises server equipped with two Tesla V100 32 GB GPUs, using Nvidia Driver 470.82 and CUDA 11.4 for efficient processing. The ADAM optimizer guided the optimization process, with Faster R-CNN trained over 20,000 iterations at a learning rate of 0.00025 and a batch size of 128, while YOLOv8 and YOLOv11 were trained over 21,140 iterations at a 0.01 learning rate with a batch size of 64. Training loss decreased and stabilized between iterations 15,000 and 20,000, indicating effective learning progress. Detection accuracy was evaluated using the intersection over union (IoU) metric, calculated by a pairwise operation within Detectron2. A detection threshold was set: bounding boxes were considered false positives if their IoU with the histopathological ground truth was less than 0.5.

### Model evaluation and statistical analysis

The multiclass image classification performance for distinguishing oral carcinoma and sarcoma in CT images on the test dataset was evaluated by precision, recall, F1 score, sensitivity, specificity, and accuracy. The accuracy performance for cancerous lesion detection of object detection models was evaluated by precision, recall, F1 score, average precision (AP), and mean average precision (mAP). Receiver operating characteristic (ROC) and precision-recall curves were generated using a Python script. An ROC curve plotted by varying the operating threshold was used to assess the ability of the classification model in the discrimination of each class. The statistical analysis for multiclass image classification and object detection was calculated as follows^21^;

$$\text{Precision}=\frac{\text{TP}}{\text{TP }+\text{ FP}}$$

$$\text{Recall }(\text{Sensitivity})=\frac{\text{TP}}{\text{TP }+\text{ FN}}$$

$$\text{F}1\text{ score}=2\text{ x }\frac{(\text{Precision x Recall})}{\text{Precision}+\text{Recall}}$$

$$\text{Specificity}=\frac{\text{TN}}{\text{TN }+\text{ FP}}$$

$$\text{Accuracy}=\frac{\text{TP}+\text{ TN}}{\text{TP}+\text{ TN }+\text{FN }+\text{ FP}}$$

where the “carcinoma” and “sarcoma” classes are positive classes and the “normal” class is a negative class.

- True Positive (TP) is the number of “carcinoma” and “sarcoma” classes that had a correct prediction or detection.
- True Negative (TN) is the number of “normal” images that had a correct prediction.
- False Negative (FN) is the number of “carcinoma” and “sarcoma” classes that had no prediction or detection.
- False Positive (FP) is the number of “normal” images that had false prediction as cancer class images.

## Results

### Multiclass image classification performance

The performance of the multiclass image classification models in distinguishing between oral carcinoma and sarcoma was evaluated using test data and is summarized in Table 1.

- **DenseNet-169**: This model demonstrated a precision of 1.0 and 0.88, a recall (sensitivity) of 1.0 and 0.98, an F1-score of 1.0 and 0.92, a specificity of 1.0 and 0.93, an accuracy of 1.0 and 0.95, and an AUC of the ROC curve of 1.0 and 0.99 for oral carcinoma and sarcoma, respectively.
- **ResNet-50**: This model demonstrated precision scores of 1.0 and 0.88, recall scores of 1.0 and 0.92, F1-scores of 1.0 and 0.90, specificity values of 1.0 and 0.94, accuracy rates of 1.0 and 0.93, and an AUC of the ROC curve of 1.0 and 0.98 for oral carcinoma and sarcoma, respectively.
- **EfficientNet-B0**: This model demonstrated a precision of 0.97 and 0.87, recall of 0.99 and 0.98, an F1-score of 0.98 and 0.92, specificity of 0.98 and 0.93, accuracy of 0.98 and 0.94, and an AUC of the ROC curve of 0.99 and 0.96 for oral carcinoma and sarcoma, respectively.
- **ConvNeXt-Base**: This model demonstrated a precision of 1.0 and 0.98, recall of 1.0 and 0.98, F1-score of 1.0 and 0.98, specificity of 1.0 and 0.99, accuracy of 1.0 and 0.99, and an AUC of the ROC curve of 1.0 and 1.0 for oral carcinoma and sarcoma, respectively.
- **ViT-Base-Patch16-224**: This model demonstrated a precision of 1.0 and 0.96, recall of 1.0 and 0.99, F1-scores of 1.0 and 0.98, specificity of 1.0 and 0.98, accuracy of 1.0 and 0.98, and an AUC of the ROC curve of 1.0 and 1.0 for oral carcinoma and sarcoma, respectively.

**Table 1:** Multiclass image classification performance metrics of the DenseNet-169, ResNet-50, EfficientNet-B0, ConvNeXt-Base, and ViT-Base-Patch16-224 regarding distinguishing oral carcinoma and sarcoma in CT images.

	Class	Precision	Recall (sensitivity)	F1 score	Specificity	Accuracy	AUC of ROC curve
DenseNet-169	Carcinoma	1.00	1.00	1.00	1.00	1.00	1.00
	Sarcoma	0.88	0.98	0.92	0.93	0.95	0.99
	Normal	0.97	0.86	0.91	0.99	0.95	1.00
						Average Accuracy = 0.97	
ResNet-50	Carcinoma	1.00	1.00	1.00	1.00	1.00	1.00
	Sarcoma	0.88	0.92	0.90	0.93	0.93	0.98
	Normal	0.92	0.88	0.90	0.96	0.93	0.98
						Average Accuracy = 0.95	
EfficientNet-B0	Carcinoma	0.97	0.99	0.98	0.98	0.98	0.99
	Sarcoma	0.87	0.98	0.92	0.93	0.94	0.96
	Normal	0.98	0.84	0.91	0.99	0.94	0.97
						Average Accuracy = 0.95	
ConvNeXt-Base	Carcinoma	1.00	1.00	1.00	1.00	1.00	1.00
	Sarcoma	0.98	0.98	0.98	0.99	0.99	1.00
	Normal	0.98	0.98	0.98	0.99	0.99	1.00
						Average Accuracy = 0.99	
ViT-Base-Patch16-224	Carcinoma	1.00	1.00	1.00	1.00	1.00	1.00
	Sarcoma	0.98	0.99	0.98	0.99	0.98	1.00
	Normal	0.97	0.96	0.97	0.98	0.98	1.00
						Average Accuracy = 0.99	

**Fig. 2:**
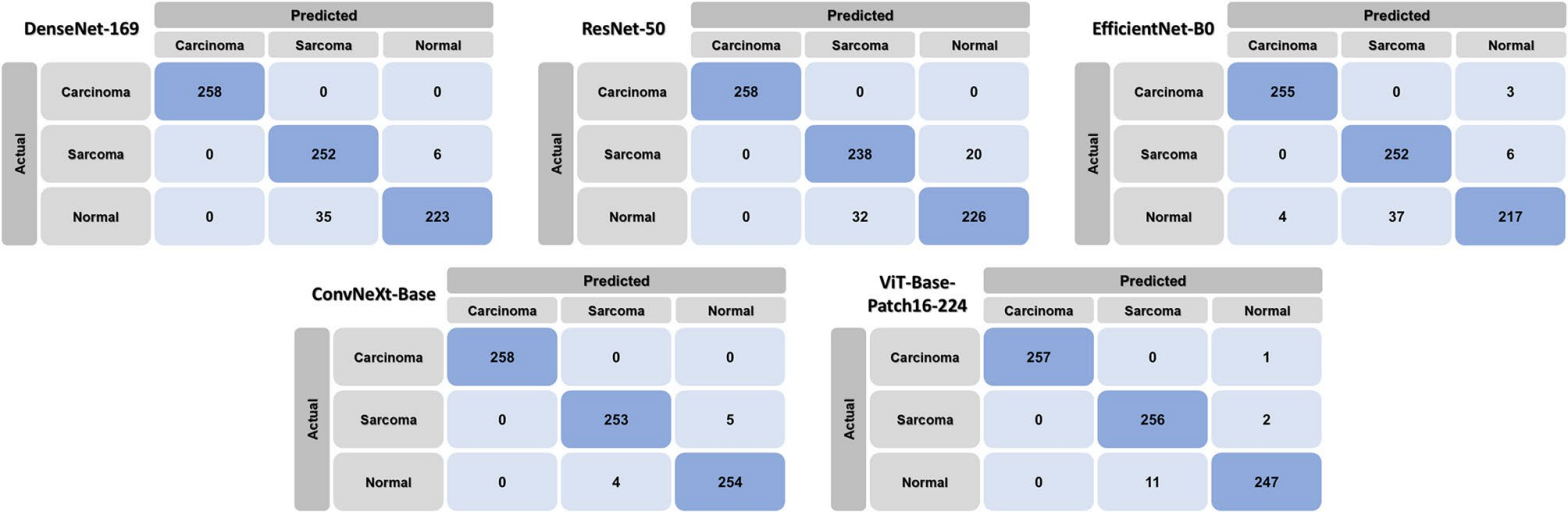
Normalized confusion matrices of image classification by DenseNet-169, ResNet-50, EfficientNet-B0, ConvNeXt-Base, and ViT-Base-Patch16-224. ‘Actual’ shows the true results; ‘Predicted’ shows the prediction of the model.

**Fig. 3:**
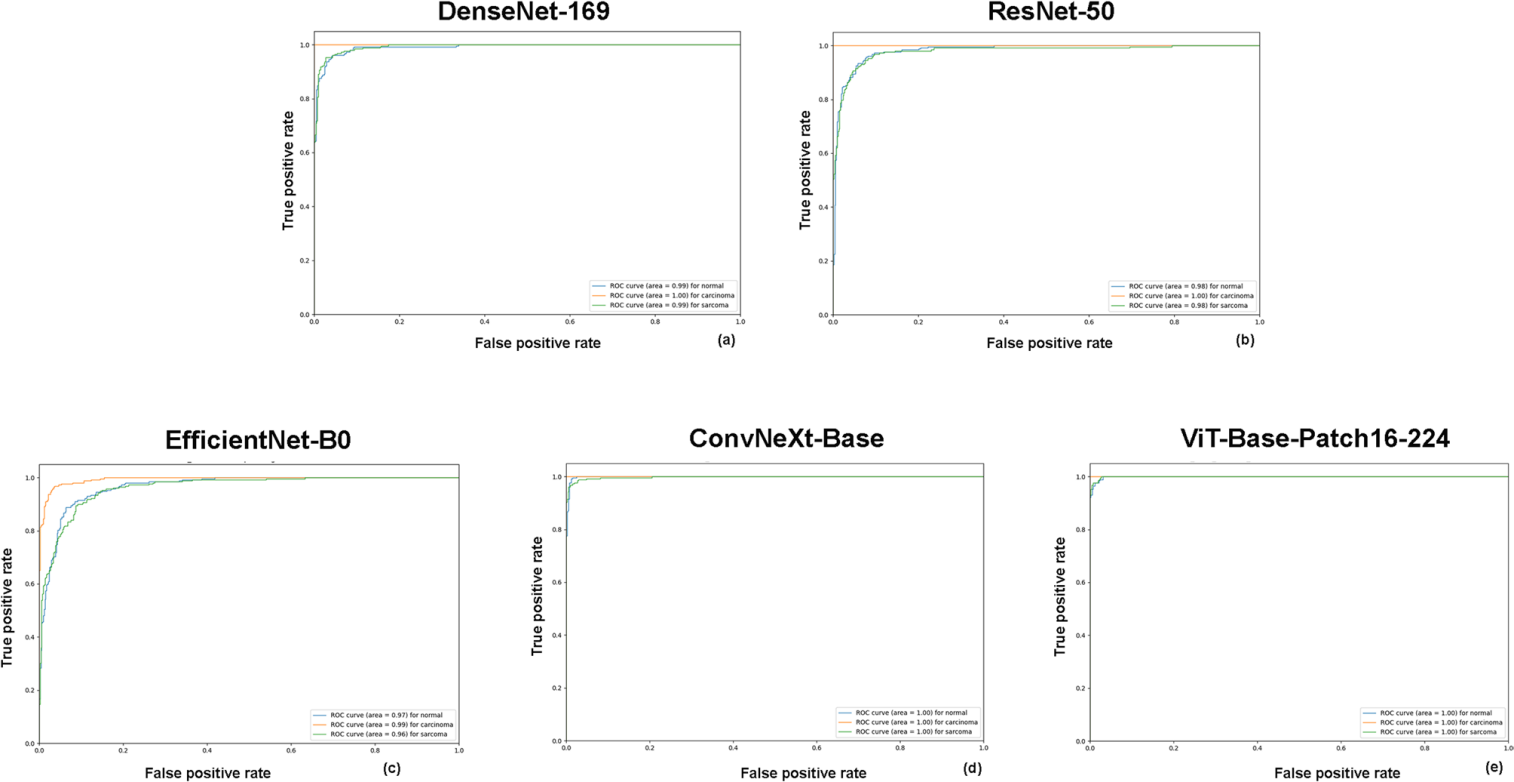
Receiver operating characteristic (ROC) curves for multiclass classification using DenseNet-169 (**a**), ResNet-50 (**b**), EfficientNet-B0 (**c**), ConvNeXt-Base (**d**), and ViT-Base-Patch16-224 (**e**). The area under the curve (AUC) values for classifying oral carcinoma and sarcoma in CT images were 1.0 and 0.99 for DenseNet-169; 1.0 and 0.98 for ResNet-50; 0.99 and 0.96 for EfficientNet-B0; 1.0 and 1.0 for ConvNeXt-Base; and 1.0 and 1.0 for ViT-Base-Patch16-224, respectively. *AUC* area under the ROC curve.

The normalized confusion matrix for each image segmentation model is shown in Fig. 2. In addition, the ROC curves for each model’s performance in classifying oral carcinoma and sarcoma are shown in Fig. 3.

### Multiclass objection detection performance

The performance of the trained object detection models in accurately detecting oral cancer lesions in the test data is summarized in Table 2.

- **Faster R-CNN**: This model demonstrated a precision of 0.65 and 0.66, a recall of 0.90 and 0.97, an F1 score of 0.76 and 0.78, and an AP of 0.80 and 0.94, for oral carcinoma and sarcoma, respectively.
- **YOLOv8**: This model demonstrated a precision of 0.88 and 0.94, a recall of 0.86 and 0.91, an F1 score of 0.87 and 0.93, and an AP of 0.83 and 0.91, for oral carcinoma and sarcoma, respectively.
- **YOLOv11**: This model demonstrated a precision of 0.87 and 0.97, a recall of 0.84 and 0.97, an F1 score of 0.85 and 0.97, and an AP of 0.82 and 0.97, for oral carcinoma and sarcoma, respectively.

**Table 2:** Multiclass object detection performance metrics of the faster R-CNN, YOLOv8 and YOLOv11 for detection of oral carcinoma and sarcoma lesions in CT images.

	Class	Precision	Recall	F1 score	AP	AUC of precision-recall curve
Faster R-CNN	Carcinoma	0.65	0.90	0.76	0.80	0.78
	Sarcoma	0.66	0.97	0.78	0.94	0.93
					mAP = 0.87	
YOLOv8	Carcinoma	0.88	0.86	0.87	0.83	0.83
	Sarcoma	0.94	0.91	0.93	0.91	0.90
					mAP = 0.87	
YOLOv11	Carcinoma	0.87	0.84	0.85	0.82	0.81
	Sarcoma	0.97	0.97	0.97	0.97	0.96
					mAP = 0.89	

*AP* average precision, *mAP* mean average precision, *AUC* area under the curve.

**Fig. 4:**
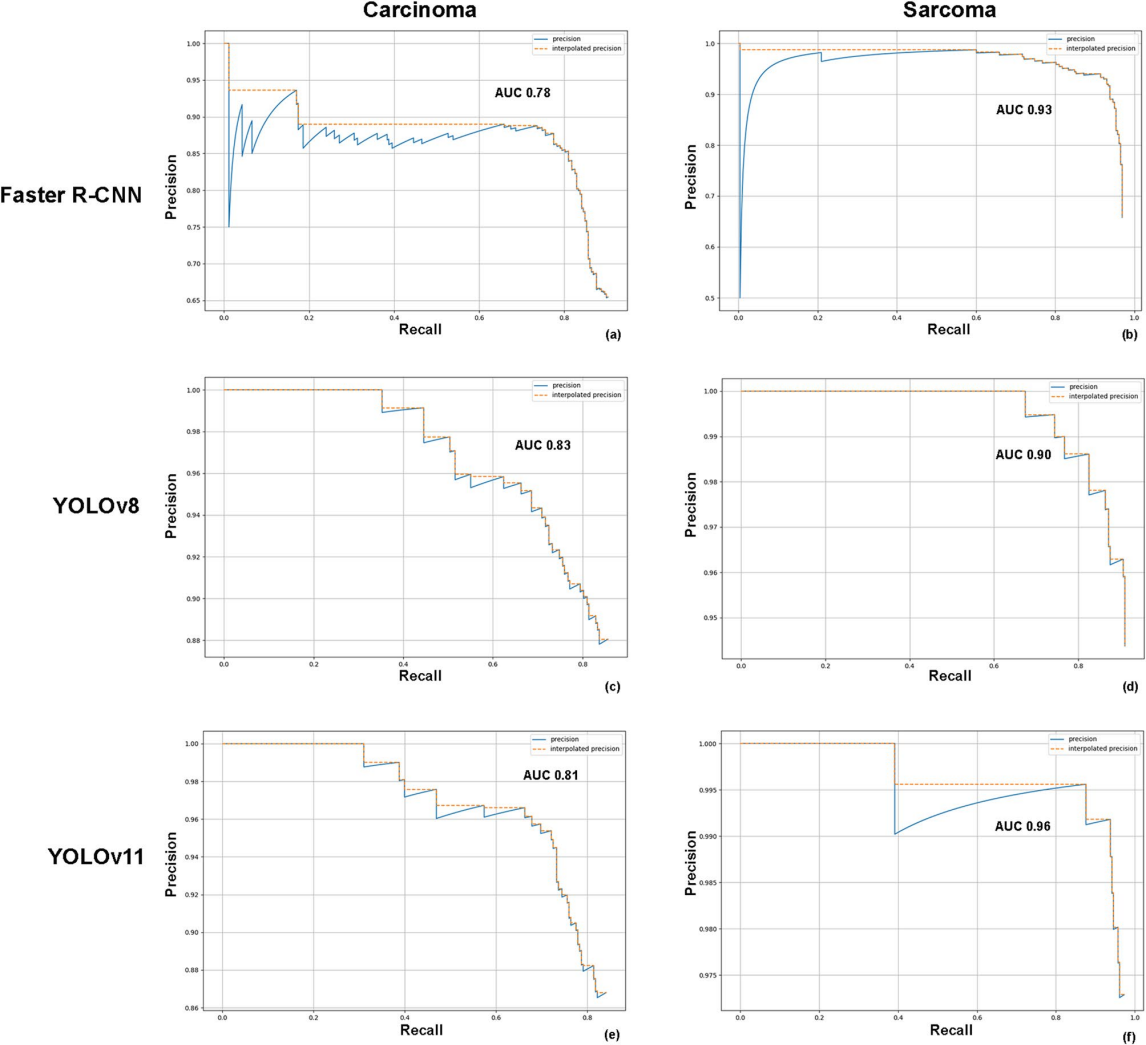
Precision-recall curves for multiclass object detection using Faster R-CNN (**a**,**b**), YOLOv8 (**c**,**d**), and YOLOv11 (**e**,**f**). *AUC* area under the precision-recall curve.

**Fig. 5:**
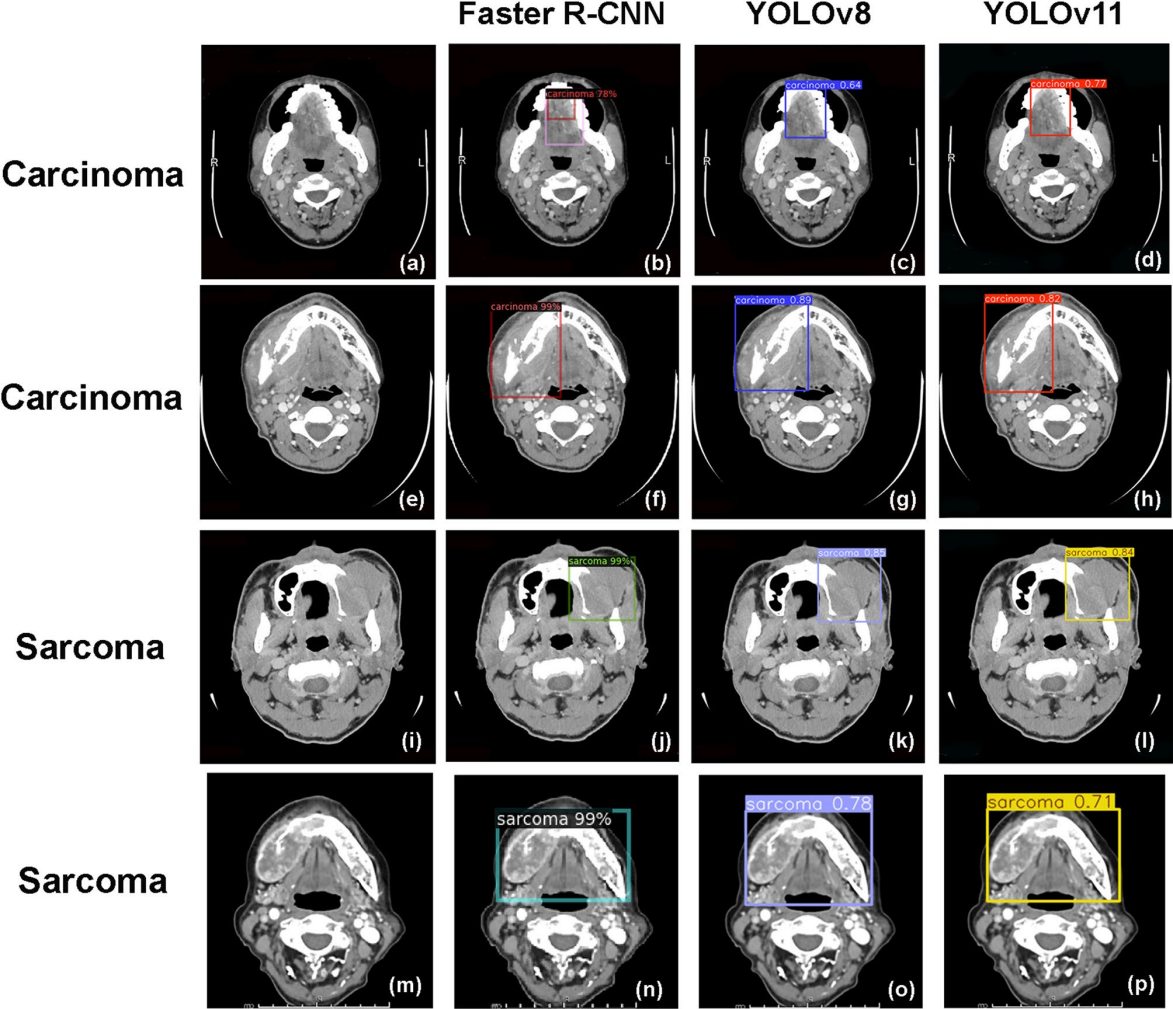
Examples of original CT images with detection bounding boxes generated by Faster R-CNN, YOLOv8, and YOLOv11 for detecting oral carcinoma and sarcoma from the test set. True positive outputs for the carcinoma class from Faster R-CNN, YOLOv8, and YOLOv11 in patients with oral squamous cell carcinoma had intersection over union (IoU) values of 0.78, 0.99, and 0.64, and 0.89, and 0.77, 0.82 respectively (**b**,**c**,**d**,**f**,**g**,**h**). True positive outputs for the sarcoma class from Faster R-CNN, YOLOv8, and YOLOv11 in patients with B-cell lymphoma had IoU values of 0.99, 0.85, and 0.99, and 0.78, and 0.84, 0.71 respectively (**j**,**k**,**l**,**n**,**o**,**p**). IoU Intersection over union.

The mAP for detecting oral cancer lesions in CT images was 0.87 for both the Faster R-CNN and YOLOv8 models. The precision-recall curves and their respective areas under the curve (AUC) for the detection of oral carcinoma and sarcoma are illustrated in Fig. 4. Examples of detection outputs from the Faster R-CNN, YOLOv8 and YOLOv11 on oral cancerous CT images are presented in Fig. 5.

## Discussion

This study demonstrated the potential of CNNs to accurately distinguish between oral carcinoma and sarcoma in contrast-enhanced CT images. Oral carcinoma and sarcoma, a type of oral cancer, are hazardous diseases and hence early detection is of utmost importance for better prognosis. The gold standard for diagnosis of oral carcinoma and sarcoma is tissue biopsy for histopathological examination, which is time-consuming^22^. Oral sarcomas mostly had a more aggressive behavior, being able to quickly invade the surrounding tissues of the oral cavity^23^. In addition to the clinical manifestations of tumors, imaging, especially contrast-enhanced CT images, can provide clues to distinguish and detect these two types of oral cancer for an effective treatment plan, particularly in the deep areas of the oral cavity, which are difficult to access for tissue biopsy. With the advancement of AI-based visualization, the CNN model could improve the visual discrimination and detection of oral carcinoma and sarcoma in CT images because of its ability to analyze complex patterns in medical data, combined with clinical data, as a diagnostic tool to assist surgeons by providing information to support decision-making in cases of oral cancer.

This study explored the application of various state-of-the-art CNN-based models, including DenseNet-169, ResNet-50, EfficientNet-B0, ConvNeXt-Base, and ViT-Base-Patch16-224, to the challenging task of multiclass classification between oral carcinoma and sarcoma in contrast-enhanced CT images. Each model showcased substantial capabilities in this domain, with DenseNet-169 and ResNet-50 achieving average accuracies of 0.97 and 0.96, respectively. EfficientNet-B0, ConvNeXt-Base, and ViT-Base-Patch16-224 achieved even higher accuracies of 0.95, 0.99, and 0.99, respectively, demonstrating their advanced capacity for feature learning and classification precision. They effectively completed carcinoma class predictions with minimal confusion between carcinoma and sarcoma features, although some errors were noted in distinguishing sarcoma from non-pathological CT images. Notably, this study is among the first to extensively evaluate and compare these advanced CNN architectures for tumors of different origins (carcinoma vs. sarcoma) in the head and neck regions using enhanced CT images. Previous works in the head and neck region in CT images have primarily focused on identifying cancerous lesions or classifying malignant and benign tumors in specific areas like the nasopharynx, oropharynx, and thyroid, often achieving sensitivities and specificities ranging from 0.5 to 0.9^24–28^. However, our work contributes novel insights by examining the unique characteristics and classification challenges of both carcinoma and sarcoma simultaneously, addressing a significant gap in the current literature. Furthermore, our comparison of classification performance in distinguishing oral tumors against previous studies utilizing non-ensemble segmentation CNNs for identifying boundaries and classifying jaw tumors^29^ revealed that our model achieved superior classification performance. This improvement may be attributed to the more distinct features of oral carcinomas and sarcomas compared to the ambiguous features typically present in jawbone tumors.

Building upon the novel classification approaches, our study also explored the detection of oral cancer lesions in CT images using CNN-based object detection models, specifically Faster R-CNN, YOLOv8 and YOLOv11. These models demonstrated high detection performance, achieving average precision (AP) scores of 0.80, 0.94, and 0.81 for oral carcinoma, and 0.83, 0.91, and 0.97 for sarcoma lesions, respectively. Interestingly, CNN-based object detection models could detect sarcomas more accurately than carcinoma lesions in CT images, which is different from classification models that could more accurately identify the class of carcinoma. This could be due to the characteristics of the object detection model which gave positive results for the interested object when the detection threshold or IoU value between the bounding box and the ground truth is greater than 0.5. Previous studies that adopted CNN-based object detection models to detect head and neck lesions in CT images reported high accuracy in tumor detection with a precision range of 0.7 to 0.9 and a recall of 0.6 to 0.9^30–32^. While detecting anomalies across multiple classes can generally reduce accuracy compared to binary detection tasks, these findings confirm that CNN-based object detection models, including Faster R-CNN, YOLOv8, and YOLOv11, exhibit substantial potential for accurate multiclass anomaly detection in CT images, reinforcing the novel applications of these models in advancing diagnostic capabilities.

In clinical practice, oral cancer patients received contrast-enhanced CT scan as a standard procedure for identifying characteristics, boundaries, and invasion of tumor^5^. Therefore, the integration of CNN-based models to CT images could assist surgeons not only in the detection of tumors to reduce human errors in misdiagnosis but also could classify the types of oral cancer as supporting information for the decision-making to enhance diagnostic confidence and consistency and support surgical planning. It is important to emphasize that while screening oral carcinomas and sarcomas using contrast-enhanced CT is beneficial, it should not replace histopathological examination as the definitive diagnostic method. Nevertheless, CT remains an invaluable tool for guiding diagnosis, assessing lesion extent, understanding tumor behavior, and supporting treatment planning.

This study had limitations that need to be declared. First, the amount of data was limited, especially oral sarcoma because of the rarity of this type of oral cancer. Second, the CT image data primarily came from three institutions, which could affect the generalizability of data. Third, the CT images in this study were contrast-enhanced CT images, so these models might not accurately analyze tumors in external test data consisting of non-contrast-enhanced CT images of the head and neck area. Due to the rarity of oral sarcomas, we did not initially distinguish between soft tissue and bone sarcomas in our analysis. However, we recognize that sarcomas include both types, each exhibiting distinct features on CT images. While soft tissue sarcomas and carcinomas can invade bone tissue, they typically do not show hyperdense or radiopaque characteristics, whereas bone or chondromatous sarcomas are classified as intra-bony lesions. To improve the accuracy and reliability of our findings, we understand the importance of establishing explicit classification guidelines to differentiate among these lesion types. This approach will help minimize potential sample selection bias and ensure our model can generalize effectively to various clinical scenarios. Future iterations of this study will prioritize clearer criteria for sample selection and careful consideration of the specific radiographic features related to each type of lesion during training and evaluation, ultimately enhancing the model’s generalization across diverse clinical situations.

The future direction of this study is to expand the dataset by including a more diverse range of head and neck CT image data through collaboration with the multi-cancer center and general hospitals. The model would be optimized for generalization by additional external data from multiple institutions to further enhance the robustness of the model. In addition, including more oral sarcoma cases to develop the AI model which could classify the oral sarcoma subtype could provide more benefits to the clinical practice of oral cancer, especially the AI model that could classify oral lymphoma, which had a different primary treatment (chemotherapy with/without radiotherapy) from other types of oral carcinoma or sarcoma that had surgery as primary treatment. To mitigate overfitting, we will implement a more stringent dataset splitting strategy to ensure that slices from the same patients do not appear across different sets. Additionally, we will explore advanced data augmentation techniques to further enhance model generalization. To reduce the risk of data contamination and improve the model’s reliability, we will use images from patients with inflammatory lesions in other head and neck regions, excluding oral involvement, as “normal” controls in future studies. Furthermore, we intend to integrate the hierarchical features of CT images to features of other factors including clinical, epidemiological, histopathological, and genomic data for multi-modelling analysis to facilitate enhanced diagnosis of oral cancer cases. While this study primarily focused on the classification task, we recognize the significant potential for integrating both classification and detection within a unified framework. Current state-of-the-art deep learning practices emphasize the development of end-to-end solutions that streamline both tasks. Therefore, the future work will explore approaches that synthesize these processes, including the adoption of joint learning models capable of simultaneously classifying and detecting anomalies in CT images. This integrated approach could provide more cohesive insights into disease characterization, ultimately enhancing the robustness and utility of diagnostic tools in clinical practice.

## Conclusion

Determination of different types of oral cancers, which are oral carcinoma and sarcoma, from CT images could support the surgeons for treatment planning to provide the best possible outcome of oral cancer cases. This study proposes highly effective CNN-based multiclass classification models that accurately distinguish between oral carcinoma and sarcoma in contrast-enhanced CT images. In particular, these classification models demonstrated the ability to completely identify oral carcinoma in CT images. In addition, the CNN-based multiclass object detection models of this study revealed the potential to accurately detect the different types of oral cancerous lesions in CT images. This work could serve as a fundamental model for the further development of oral cancer screening applications to discriminate between types of oral cancer in CT imaging. It could also benefit oral cancer cases that appear in the deep parts of the oral cavity and are difficult to access for tissue biopsy, providing supporting information for surgeons to select the most appropriate treatment plan. Continued advancements in CNN technology provide the potential to significantly improve diagnostic accuracy, support treatment planning, and ultimately increase survival rates for oral cancer cases.

## Data Availability

The datasets used and analyzed during the current study are available from the corresponding authors on reasonable request.
